# Shape Matters: The Utility and Analysis of Altered Yeast Mitochondrial Morphology in Health, Disease, and Biotechnology

**DOI:** 10.3390/ijms26052152

**Published:** 2025-02-27

**Authors:** Therese Kichuk, José L. Avalos

**Affiliations:** 1 Department of Molecular Biology, Princeton University, Princeton, NJ 08544, USA; tkichuk@princeton.edu; 2 Rutgers Robert Wood Johnson Medical School, New Brunswick, NJ 08901, USA; 3 Department of Chemical and Biological Engineering, Princeton University, Princeton, NJ 08544, USA; 4 Omenn-Darling Bioengineering Institute, Princeton University, Princeton, NJ 08544, USA; 5 The Andlinger Center for Energy and the Environment, Princeton University, Princeton, NJ 08544, USA; 6 High Meadows Environmental Institute, Princeton University, Princeton, NJ 08544, USA

**Keywords:** mitochondria, morphology, fission, fusion, contact sites, pathology, neurodegenerative, cancer, biofuel, engineering, imaging, analysis

## Abstract

Mitochondria are involved in a wide array of critical cellular processes from energy production to cell death. The morphology (size and shape) of mitochondrial compartments is highly responsive to both intracellular and extracellular conditions, making these organelles highly dynamic. Nutrient levels and stressors both inside and outside the cell inform the balance of mitochondrial fission and fusion and the recycling of mitochondrial components known as mitophagy. The study of mitochondrial morphology and its implications in human disease and microbial engineering have gained significant attention over the past decade. The yeast *Saccharomyces cerevisiae* offers a valuable model system for studying mitochondria due to its ability to survive without respiring, its genetic tractability, and the high degree of mitochondrial similarity across eukaryotic species. Here, we review how the interplay between mitochondrial fission, fusion, biogenesis, and mitophagy regulates the dynamic nature of mitochondrial networks in both yeast and mammalian systems with an emphasis on yeast as a model organism. Additionally, we examine the crucial role of inter-organelle interactions, particularly between mitochondria and the endoplasmic reticulum, in regulating mitochondrial dynamics. The dysregulation of any of these processes gives rise to abnormal mitochondrial morphologies, which serve as the distinguishing features of numerous diseases, including Parkinson’s disease, Alzheimer’s disease, and cancer. Notably, yeast models have contributed to revealing the underlying mechanisms driving these human disease states. In addition to furthering our understanding of pathologic processes, aberrant yeast mitochondrial morphologies are of increasing interest to the seemingly distant field of metabolic engineering, following the discovery that compartmentalization of certain biosynthetic pathways within mitochondria can significantly improve chemical production. In this review, we examine the utility of yeast as a model organism to study mitochondrial morphology in both healthy and pathologic states, explore the nascent field of mitochondrial morphology engineering, and discuss the methods available for the quantification and classification of these key mitochondrial morphologies.

## 1. Introduction

The study of mitochondria and mitochondrial genetics is equally rooted in *S. cerevisiae* and serendipity. As important as the scientific method is for proving or disproving a hypothesis, a great many scientific discoveries begin by chance. Serendipity was in full force the day the young Polish rebel Piotr Slonimski found a book containing the work of Russian-born geneticist Boris Ephrussi in the ruins of a German police station during World War II. The genetic studies detailed in the book were so profoundly inspiring to Slonimski that he moved to Paris to work with Ephrussi, and together, they established a system in *S. cerevisiae* to study the mitochondrial genome [1].

As Ephrussi and Slonimski demonstrated, yeast is particularly suited to the study of mitochondria due to its ability to grow anaerobically. Where mammalian cells would die, yeast can survive completely lacking mitochondrial genomes and, although these respiratory-deficient cells exhibit abnormal mitochondrial morphologies, they are viable [2]. Furthermore, because yeast and mammalian mitochondria are highly conserved, yeast has long been used as a proxy to facilitate studies of human mitochondrial processes [3]. The ability to delete and mutate the nuclearly or mitochondrially encoded mitochondrial genes involved in respiration has proven invaluable for both studies of human disease and yeast strain engineering.

It is difficult to overstate the importance of mitochondria in eukaryotic cell biology. In fact, it is widely accepted that the endosymbiotic event leading to the origin of mitochondria was a pivotal moment in the history of life on Earth, as mitochondria in their earliest form provided the substantial energy necessary for the evolution of the eukaryotic cell [4]. Mitochondria are critical not only for energy production, but also key metabolic pathways, calcium homeostasis, and even apoptotic cell death. Considering the variety of intracellular roles these organelles play, perhaps it is no surprise that mitochondrial networks dynamically respond to changes in the cellular environment [5]. Yeast growing in glucose will mostly rely on fermentation for energy production, while the same yeast grown in solely glycerol or ethanol media will grow exclusively by respiration, using oxidative phosphorylation to produce ATP (Table 1). While cells are fermenting, the mitochondrial networks are tubular and distributed asymmetrically throughout the cell, whereas respiring cells have highly branched, predominately peripheral mitochondrial networks [6,7]. In this way, mitochondrial morphologies reflect the metabolic state of the cell, and the size and shape of these organelles can serve as valuable indicators of both cellular function and dysfunction.

Mitochondrial morphology is governed by the constant interplay between fission and fusion in addition to the balance between mitochondrial biogenesis and mitophagy (mitochondrial recycling) [8]. The molecular machinery required for fission, fusion, and mitophagy is found at mitochondria–endoplasmic reticulum (mito-ER) junctions [9,10]. Mitochondrial dynamics, therefore, are impacted by interactions with other subcellular compartments such as the endoplasmic reticulum (ER), also influencing [8,9,10] mitochondrial shape, number, and volume.

Abnormal mitochondrial morphologies and altered inter-organelle interactions have become hallmarks of a variety of disease states such as Parkinson’s disease and cancer. Interestingly, increased mitochondrial fission is a key factor in both diseases. In Parkinson’s disease, excessive mitochondrial fission produces fragmented mitochondria with compromised capacity for energy production, ultimately resulting in neuronal death [11,12]. This observation suggests that excessive mitochondrial fission is detrimental to cell viability. Numerous cancers, however, are also characterized by increased mitochondrial fission [13,14,15]. The fragmented mitochondrial morphologies in cancer cells are credited with allowing cancer cells to meet the high energy demands of their hyperproliferative states and resist cell death. These opposite cell fates related to apparently the same change in mitochondrial morphology illustrate the significant gaps that still exist in our understanding of the role of mitochondrial morphology in human health and disease.

While abnormal mitochondrial morphologies might lead to several devastating pathologies, recent studies in organelle and metabolic engineering explore whether altering mitochondrial shapes can be exploited to develop strains for chemical production [16,17]. It has been shown that engineering mitochondrial size and number can benefit mitochondrially localized biosynthetic pathways, likely by constraining the cytoplasmic spread of enzymes and substrates by containing them in smaller compartments. One study has demonstrated an increase in mitochondrially localized sabinene production when genes governing mitochondrial morphology, like fission gene *FIS1,* are over-expressed [16]. Another study showed increased isobutanol production from mitochondria with smaller volumes [17]. This work has been performed largely in the baker’s yeast *S. cerevisiae*, the very organism that originated genetic studies of mitochondrial function. Therefore, complementarities can be built between the fields of mitochondrial pathophysiology and metabolic engineering, centering on studies of yeast mitochondrial metabolism, morphology, and dynamics.

The importance of altered mitochondrial morphology across fields has underscored how crucial it is to characterize mitochondrial phenotypes and dynamics accurately and quantitatively. Enhanced techniques have allowed unprecedented image resolution, leading to much improved data. Of equal importance, novel analytical methods have provided a more precise vocabulary to describe mitochondrial morphologies. One of the greatest advances in image analysis in the past several decades has been the rise of three-dimensional rendering, which makes it possible to recreate and rotate objects of interest in silico and analyze them from all angles. For investigating frozen cell samples, electron tomography offers unparalleled resolution from 2 to 20 nm in three dimensions [18,19,20]. For live cell imaging, fluorescence confocal microscopy images can be analyzed with open-source programs like Fiji or three-dimensionally rendered for more comprehensive analysis with the open-source program MitoGraph or commercially available software such as Arivis, or Amaris [21,22]. In this review, we discuss the implications of altered mitochondrial morphologies in health and disease, the potential to engineer these morphologies to improve yeast strains for metabolic engineering, and the tools with which we can quantify and study these morphological variations.

## 2. Processes Governing Yeast Mitochondrial Morphology

**Figure 1:**
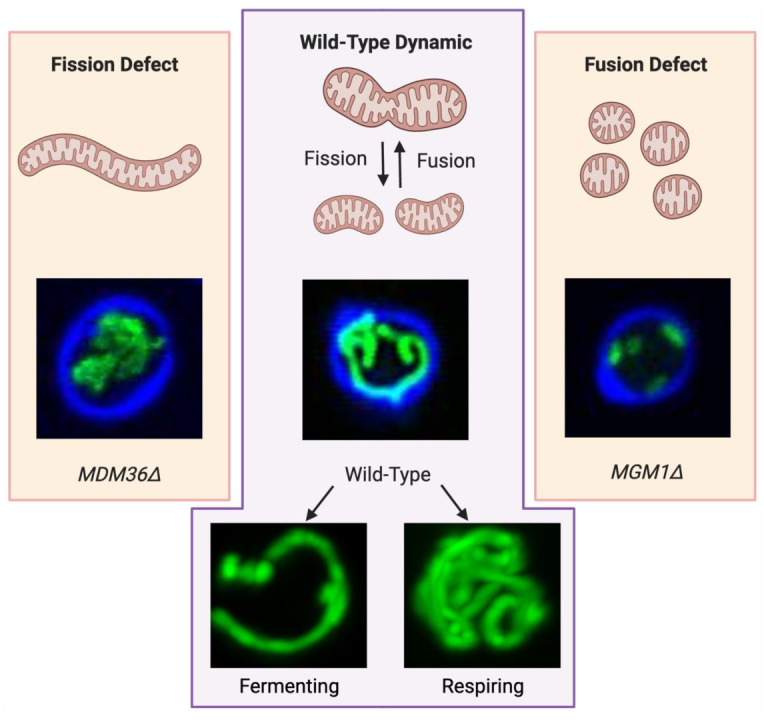
Mitochondrial morphology is the result of a balance between fission and fusion processes and responds to the cell’s metabolic state.

Mitochondria are highly dynamic organelles with morphologies that respond to their environment through a balance of mitochondrial fission, fusion, mitophagy, and biogenesis. In the yeast *S. cerevisiae*, randomly distributed mitochondrial tubules morph into highly branched, peripherally located mitochondrial networks when the cells transition from fermentation to respiration (Figure 1) [6,23]. When the balance of fission and fusion is disrupted, such as by deleting a gene crucial to either process, mitochondrial morphology is impacted. Cells with defective fission will demonstrate single globular mitochondria, whereas cells with deficient fusion will exhibit multiple small mitochondrial compartments. The balance of both processes is essential for normal mitochondrial form and function [7,9,24].

During fermentation, yeast mitochondria (marked in green) exhibit tubular morphologies whereas during respiration, mitochondria form highly branched networks that provide greater surface area. Fission defects such as those in MDM36Δ strains result in aggregated mitochondrial phenotypes whereas fusion defects such as those in MGM1Δ strains result in fragmented and punctate morphologies. Green fluorescent protein fused to a mitochondrial localization signal is used to visualize mitochondria (green), while calcofluor white is used to visualize the cell wall (blue in top pictures). The MDM36Δ and MGM1Δ strains are shown growing in fermentative conditions, while the wild-type is shown transitioning from fermentation to respiration [6].

**Figure 2:**
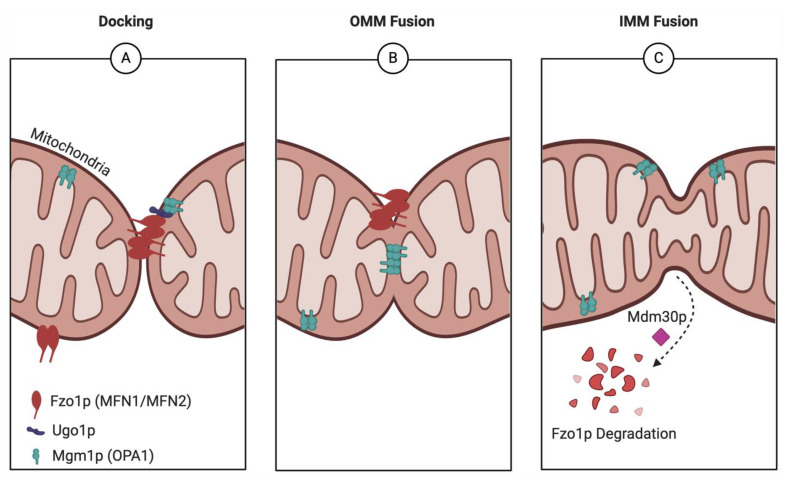
Yeast mitochondrial fusion. (**A**) Fzo1p homodimerizes on the OMM and associates with other Fzo1p homodimers on neighboring mitochondria, tethering the two mitochondrial compartments together. Meanwhile, Ugo1p acts as a bridge between on Fzo1p and Mgm1p assemblies in the OMM and IMM, respectively, to coordinate the outer and inner mitochondrial membrane fusion machinery. (**B**) Following fusion of the OMM, Mgm1p catalyzes the fusion of the IMM. (**C**) Hydrolysis of GTP by Fzo1p induces a conformational change in these homodimeric complexes that allows for Fzo1p ubiquitylation by the F-box protein Mdm30p, ultimately leading to Fzo1 degradation and clearance, which maintains steady-state levels of Fzo1p.

Mitochondrial fusion involves the sequential joining of the mitochondrial outer membrane (OMM) and the mitochondrial inner membrane (IMM) to merge two previously separate mitochondrial compartments [25] (Figure 2). In *S. cerevisiae*, mitochondrial fusion is mediated primarily by three different nuclearly encoded proteins, Fzo1p, Mgm1p, and Ugo1p [26]. Yeast OMM fusion is initiated by Fzo1p (Fuzzy onion 1 protein), which is the yeast ortholog of the mammalian mitofusins (MFN1 and MFN2). Fzo1p homo-dimerizes on the OMM and associates with other Fzo1p homodimers on neighboring mitochondria, tethering the two mitochondrial compartments together (Figure 2A). The GTPase activity of Fzo1p then fuels the fusion of the OMMs of neighboring mitochondria. In a similar process, the dynamin-related GTPase Mgm1p forms homodimer assemblies in the mitochondrial inner membrane (IMM), which mediate IMM fusion after the OMMs have fused (Figure 2B) [27]. Mgm1p is functionally equivalent to the mammalian protein OPA1, a key regulator of IMM fusion in mammalian cells [28]. The third protein key to yeast fusion is Ugo1p. This protein forms a complex with Mgm1p and Fzo1p and is thought to be responsible for coordinating the OMM and IMM fusion mechanisms (Figure 2A) [29]. Though the mechanism of Ugo1p is not completely understood, deletion of the human Ugo1p homolog SLC25A46 alters the mitochondrial lipid profile and the oligomerization states of OPA1 and MFN2 [30,31]. The current hypothesis is that Ugo1p and its human homolog mediate lipid flux from the ER to the mitochondria, to create an optimal lipid environment for GTPase oligomerization.

Steady-state levels of Fzo1p are crucial to maintaining intact fusion processes and additional proteins such as Mdm30p help control mitochondrial fusion by preventing unregulated accumulation of Fzo1p [32,33]. Hydrolysis of GTP by Fzo1p induces a conformational change in Fzo1p homodimeric complexes, allowing for Fzo1p ubiquitylation by the F-box protein Mdm30p. This ubiquitylation ultimately leads to Fzo1p degradation and clearance [32,33], while Mgm1p is thought to remain in the intermembrane space to facilitate cristae formation and maintenance [34] (Figure 2C). Notably, essential fusion proteins such as Fzo1p and Mgm1p are also crucial to maintaining the mitochondrial genome [35,36]. Yeast null mutants of *FZO1*, *MGM1*, and *UGO1* exhibit unusually fragmented mitochondria and defective respiration due to mitochondrial DNA (mtDNA) loss.

**Figure 3:**
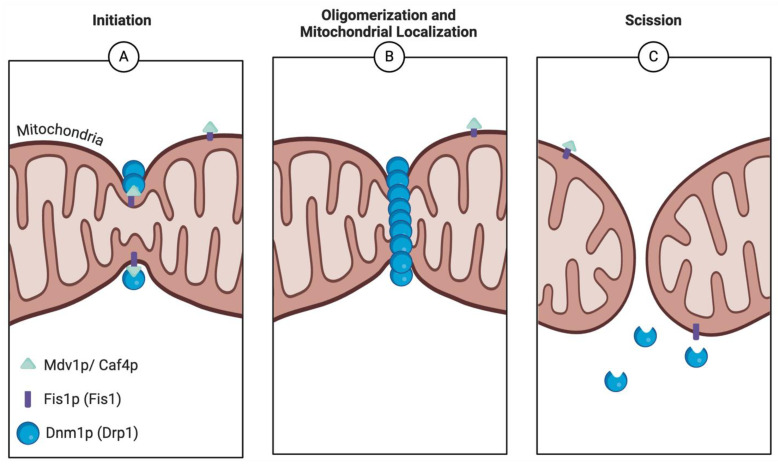
Yeast mitochondrial fission. (**A**) Dnm1p is recruited to the surface of the mitochondria by Fis1p, which is anchored to the OMM, through adapter proteins such as Mdv1p. (**B**) On the surface, Dnm1p collects and self-assembles into ring-like structures around the mitochondrial tubule, leading to (**C**) constriction and the formation of a site for scission.

The counterpoint to mitochondrial fusion is mitochondrial fission, in which the mitochondrion splits, creating multiple smaller mitochondria (Figure 3). The excessive fragmentation of mitochondria observed in *FZO1*, *UGO1*, and *MGM1* mutants is a result of continuous mitochondrial division without the balance of intact fusion processes [37,38]. In *S. cerevisiae*, mitochondrial division is facilitated by Dnm1p, Mdv1p, and Fis1p [38,39,40,41]. Dnm1p (Drp1 in humans) is a large cytoplasmic GTPase, which is recruited by OMM-anchored Fis1p through the adapter proteins Mdv1p and Caf4p to the surface of the mitochondria (Figure 3A). On the surface, Dnm1p collects and self-assembles into ring-like structures (Figure 3B) around the mitochondrial tubule. GTP hydrolysis then leads to a conformational shift resulting in mitochondrial constriction and formation of a site for scission (Figure 3C). In human cells, further mitochondrial constriction and eventual scission are catalyzed by GTPase dynamin 2 (Dnm2) though the yeast homolog Vps1p has not been demonstrated to have a significant role in mitochondrial fission [42,43]. Yeast mutants lacking Dnm1p, Mdv1p, and Fis1p are unable to undergo fission and therefore exhibit a single mitochondrion composed of condensed and interconnected compartments. Double deletions of both fission and fusion essential genes, such as *FZO1/DNM1, UGO1/DNM1,* and *MGM1*/*DNM1*, however, display quasi-wild-type tubular mitochondria but demonstrate impaired respiratory capacity. This indicates that while the regulation of mitochondrial shape and number is determined by the balance between fusion and fission, mitochondrial processes like respiration depend on intact fission and fusion processes [38,44,45].

Interactions between mitochondria and other organelles play a crucial role in regulating mitochondrial morphology in both yeast and mammalian cells. In yeast, mitochondria closely interact with the ER through specialized contact sites known as the ER-mitochondria encounter structure (ERMES) [46]. The ERMES complex in yeast is made up of four proteins: Mdm10p, Mdm12p, Mdm34p, and Mmm1p. These proteins interact to form a molecular anchor tethering the ER to the OMM and facilitating the exchange of lipids and other molecules between the two organelles. Similarly, the ER also forms contact sites with mitochondria in mammalian cells through what is known as mitochondria-associated ER membranes (MAMs) [24]. These contact sites play a crucial role in lipid metabolism, calcium homeostasis, and the regulation of mitochondrial dynamics. In mammalian systems, MAMs are regulated by MFN2 (mitofusin 2) and form stable contacts between the mitochondria and ER to allow for inter-organelle crosstalk [47,48]. MAMs consist of calcium channels that modulate mitochondrial calcium levels to impact both cellular metabolism and autophagy [49,50,51]. In both mammalian and yeast cells, mitochondrial fission and fusion occur at mito-ER junctions [9], and their distribution is influenced by the actin cytoskeleton [52]. In budding yeast, actin filaments provide the molecular railroad upon which mother cells pass along mitochondria to daughter cells [53]. While mitochondrial movement in mammalian cells is largely driven by microtubules [54], actin plays an important role in mitochondrial retention in neuronal areas with high ATP usage [55]. Mitochondrial morphology in yeast and mammalian cells is also influenced by contact with other cellular compartments such as lipid droplets (LDs), which serve as sites of lipid storage and metabolism [56,57]. In brown adipose cells, mitochondria associated with LDs have reduced fission and fusion dynamics to support distinct mitochondrial bioenergetics [56]. In yeast, LDs protect mitochondria from excessive fission induced by aging [57]. In this way, numerous subcellular structures impact mitochondrial morphology and position within the cell.

Interactions between organelles can also have a profound influence on mitochondrial degradation. Mito-ER contacts are crucial to mitophagy, the selective degradation and recycling of mitochondria by autophagy. By tethering mitochondria to the ER, ERMES promotes the formation of mitophagosomes (double-membrane vesicles that engulf mitochondria for degradation) and facilitates the selective autophagic elimination of mitochondria [10]. In mammalian cells, MAMs are also implicated in the regulation of mitophagy through both PINK/Parkin-dependent mitophagy and FUNDC1-Mediated mitophagy [51]. Under normal physiological conditions, the phosphatase and tensin homolog (PTEN)-induced putative kinase 1 (PINK1) is transported into the mitochondria using the mitochondrial membrane potential where it is then degraded by presenilin-associated rhomboid-like protein (PARL) and cleared from the outer mitochondrial membrane. In the presence of mitochondrial damage, however, PINK1 cleavage is impaired and uncleaved PINK1 accumulates on the outer mitochondrial membrane. The accumulated PINK1 indirectly recruits Parkin to the OMM where it subsequently phosphorylates and activates it. In turn, Parkin polyubiquitinates many OMM proteins, which then recruit autophagosome machinery to the mito-ER junctions [58]. MAMs also contribute to the regulation of mitophagy by acting as sites of FUN14 domain containing 1 (FUNDC1) accumulation. The FUNDC1 collected at mito-ER junctions in hypoxic mammalian cells then recruits mitophagy machinery [59]. In this way, mitochondrial contact with the ER is not only crucial for morphology maintenance, but these contacts also initiate the degradation of damaged mitochondria.

While mitochondrial components can be recycled, they can also be built from scratch through the classic DNA to protein pipeline. Mitochondrial biogenesis is coordinated by both nuclearly and mitochondrially encoded genes which produce the functional components of the mitochondrial compartment. Much like in mammalian cells, most mitochondrial genes in yeast are encoded in the nucleus. Yeast mitochondrial DNA exists as a small circular chromosome encoding for a handful of genes including seven proteins involved in oxidating phosphorylation and one mitochondrial ribosomal protein [60]. Similarly, all thirteen protein products encoded for by mammalian mitochondrial DNA code for complexes involved in oxidative phosphorylation [61,62,63]. Considering the various roles of mito-ER contacts, it is no surprise that crosstalk between the mitochondria and ER also has a role in yeast mitochondrial biogenesis. As the ability of mitochondria to synthesize lipids is limited, mitochondrial biogenesis relies on the ER to supply phospholipids for future mitochondrial membrane production [64]. Not only are mito-ER contact sites a major site of phospholipid production, but they also appear to be responsible for the transport of lipids into the growing mitochondrion [65,66,67].

Through the varied and complex processes of fission, fusion, mitophagy, and biogenesis, cells regulate highly dynamic mitochondrial networks. Due to the complexity of this delicate balance, errors or disruptions to these processes caused by mutation or other dysfunctions, are often manifested in aberrant mitochondrial phenotypes. Therefore, altered mitochondrial morphologies can not only serve as indicators of physiological or metabolic perturbations, such as the transition between fermentation and respiration, but dysregulation of mitochondrial morphology is also a hallmark of numerous disease processes that can be effectively studied in yeast models.

## 3. Using Yeast to Study Mitochondrial Morphology in Human Disease

Altered mitochondrial morphologies are trademark characteristics of a wide range of human diseases. From neurodegeneration to metabolic disorders to cancer, abnormal mitochondrial phenotypes are varied in both their causes and effects on the cell. Studies of yeast mitochondria have played a role in our understanding of each of these pathologies, proving repeatedly the utility of this model organism.

It may come as a surprise due to their obvious lack of neurons, but yeast mitochondrial morphology has been particularly useful to the study of neurodegeneration. In Parkinson’s disease, excessive mitochondrial fission leads to fragmented mitochondria and compromised energy production resulting in neuronal death [11,12]. In a yeast model, human α-synuclein, a key protein that accumulates in Parkinson’s disease, was shown to trigger mitochondria-independent apoptosis, suggesting that organized cell death can be initiated in the presence of α-synuclein even without functional mitochondria, which could help us understand the pathophysiology of Parkinsonian neurodegeneration [68]. Recently, yeast models have also been used to investigate the stabilization of PINK1 at the translocase of the outer membrane (TOM) complex. By expressing human PINK1 and TOM proteins in *Saccharomyces cerevisiae*, researchers were able to clarify the role of each of the seven TOM subunits [69]. A study investigating the impact of inter-organelle contact found that mitochondrial contact with the vacuole but not the ER was necessary for α-synuclein toxicity [70].

Impaired mitochondrial fusion has also been evident in Alzheimer’s disease models, which has resulted in both increased mitochondrial fragmentation and increased mitochondrial-ER contacts [71,72,73]. Yeast models have helped demonstrate the mitochondrial toxicity of pathological amyloid-β peptides (Aβ) aggregates in Alzheimer’s disease [74] and a new *Yarrowia lipolytica* yeast model expressing a major form of Aβ (Aβ42) demonstrated increased mitochondrial fragmentation, production of reactive oxygen species, and cell death [75]. Yeast models have also been used to investigate how autophagy modulates the accumulation and aggregation of Aβ [76] and to screen drugs for possible treatments for Alzheimer’s disease [77]. A recent study on the mechanism of action of the potential Alzheimer’s therapeutic clioquinol demonstrated that it reduced Aβ42 toxicity in yeast cells by alleviating reactive oxygen species generation and the level of lipid peroxidation [78]. From uncovering potential pathological mechanisms to discovering potential treatments, the yeast mitochondrion continues to be an invaluable model to study human neurodegeneration.

Altered mitochondrial morphology is also a common feature in human disorders related to mitochondrial fusion or fission. These disorders are particularly suited for yeast studies as many of the human proteins involved have orthologs in yeast. For example, mutations in human genes MFN2 and OPA1, involved in mitochondrial fusion, lead to abnormal mitochondrial morphology and are associated with Charcot–Marie–Tooth disease and optic atrophy, respectively [79,80]. The yeast ortholog and human proteins have been fused to form functional chimeras to better understand the molecular underpinnings of these diseases [81]. The yeast/human protein chimera of Mmg1p/OPA1 was used to study the effects of missense mutations associated with optic atrophy [81]. While fewer diseases have been linked to mitochondrial fission defects, mutations in DRP1 are associated with encephalopathy with optic atrophy [82]. Yeast has been used to study these fission defects by employing the homolog Dnm1p to replicate disease-causing mutations and investigate altered mitochondrial dynamics [83].

Cancer is another group of pathologies in which altered mitochondrial morphology is a hallmark. Numerous cancers are characterized by increased mitochondrial fission due to an imbalance of MFN2/DRP1 expression [13,14,15]. The abnormal mitochondrial morphologies allow cancer cells to meet their high energy demands and contribute to metabolic reprogramming, resistance to cell death, and tumor progression. Despite its relative simplicity, yeast can also be used as a model organism to study mitochondrial dysfunction in cancer. *S. cerevisiae* has been used to improve our understanding of the role of Rat Sarcoma Virus (RAS) proteins in tumorigenesis [84] and these eukaryotes are well suited to study the Warburg effect in which tumor cells shift their primary metabolism from respiration to anaerobic glycolysis and fermentation [85].

Changes in mitochondrial morphology are also closely linked to metabolic diseases such as diabetes and cardiac disorders [86,87]. In insulin resistance and type 2 diabetes, alterations in mitochondrial dynamics, including increased fission relative to fusion, have been observed in insulin-sensitive tissues like skeletal muscle [88]. Additionally, there is evidence of mitochondrial fragmentation and increased fission in heart failure, which could result in compromised energy production in cardiac muscle cells [89]. While yeast has not yet been used as a model organism for these diseases, it has been used to investigate the cellular impact of metformin, a drug commonly used in the treatment of type 2 diabetes. This recent study found that *Schizosaccharomyces pombe* cells treated with metformin and grown in media both with and without glucose experienced longer chronological lifespans relative to cells not treated with metformin [90]. This hints at a non-glucose dependent mechanism for the positive metabolic impact of metformin treatment.

Despite the widespread use of yeast as a model organism for human disease, there are some limitations to its utility. While yeast share many molecular similarities with mammalian systems, there are fundamental distinctions between yeast and human subcellular pathways. For example, in yeast, the process of β-oxidation, the breakdown of fatty acids, occurs predominantly in peroxisomes [91,92]. This same process in mammalian cells occurs both in mitochondria and peroxisomes and therefore studies altering β-oxidation in yeast would need to account for the multiple compartments housing this pathway in mammalian systems. Additionally, in yeast, mitochondria tether to both the ER and the vacuole [93,94], while human cells lack formal vacuoles. There are also differences between the functions of these contact sites between yeast and mammalian systems. Mitochondrial contact with the ER is responsible for calcium flux into mitochondria in mammalian systems [95,96] but not in yeast. This difference is particularly important when investigating neurodegeneration, and cell death as calcium flux from the ER is part of the apoptosis cascade. Beyond the simple biological differences, studies in yeast are limited to the analysis of single cells as opposed to the whole tissue studies possible with more complex organisms. Differences aside, the relative simplicity and genetic tractability of yeast as a model organism have maintained their relevance to the study of human health and disease.

Understanding the processes governing mitochondrial morphologies in human diseases is crucial for developing new therapies. Considering the vast array of diseases associated with excessive mitochondrial fragmentation, accurately characterizing mitochondrial phenotypes is of critical importance. Accurate classification and quantification of mitochondrial phenotypes could help parse the differences between disease states and help answer paradoxical questions such as how excessive fragmentation can lead to impaired energy production in one cell [11,12], but enhanced energy production in another [13,14,15]. The tractability and hardiness of yeast, as well as the close similarity of its mitochondria to those of mammalian cells, will continue to make this fungus a useful model organism to study numerous human diseases associated with mitochondrial dysfunction.

## 4. Yeast Mitochondrial Engineering for Chemical Production

While aberrant mitochondrial morphology is a hallmark of many pathologies, dysregulation of yeast mitochondrial morphology has relevance beyond the study of human disease. Altering mitochondrial phenotypes in engineered yeast strains can be an effective strategy for increasing chemical production. Mitochondrial compartmentalization strategies have been employed to produce numerous classes of chemicals [97]. Mitochondrially targeted enzymes have enhanced alcohol production, boosting titers of both isobutanol, isopentanol, and 2-methyl-1-butanol in *S. cerevisiae* [98,99,100]. Mitochondrial compartmentalization strategies have also improved the production of terpenoids. By exploiting the physical segregation of mitochondrially localized enzymes from the rest of the cell, researchers have enhanced the production of geraniol [101] and sabinene [16]. Recent studies on squalene production have investigated the benefits of simultaneous cytoplasmic and mitochondrial engineering [102]. The increased availability of acetyl-CoA and NADPH within mitochondria has also been exploited to enhance the production of 3-hydroxypropionate (3-HP) [103]. Isolating biosynthetic pathways from the cytosolic environment decreases the potential impact of toxic intermediates, as well as the loss of valuable intermediate molecules to competing pathways. Furthermore, containing specialized enzymes within a subcellular compartment increases their local concentration and substrate availability [100]. Researchers have demonstrated a boost in chemical production by localizing enzymes to a subcellular compartment, away from the molecular chaos of the cytoplasm. Now, what if that compartment changed shape, size, or number?

In budding yeast, the overall size of the mitochondrial compartment occupies between 4.8% and 35.4% of the total yeast cell volume, depending on factors such as carbon source and aeration conditions [104]. In addition to the convenient sizing of this subcellular compartment, the dynamic nature of yeast mitochondria makes these organelles interesting engineering targets. Altering mitochondria size and shape has the potential to further increase the production of valuable chemicals. A recent paper investigating the impact of mitochondrial-related genes on the production of sabinene expressed a truncated sabinene synthase in both the cytosol and the mitochondria and found this significantly increases sabinene production [16]. Moreover, individually overexpressing four different genes (*FIS1, LSB3, MBA1,* and *AIM25*) related to a range of mitochondrial functions impacting their morphology, including a gene related to fission (*FIS1)*, further improved sabinene titers. Integrating all of these engineered genes into a host chromosome resulted in sabinene titers of 154.9 mg/L, a nearly 60-fold increase in production over the original unmodified strain. Another recent study focused on the effects that deletions of genes involved in mitochondrial fission and fusion have on the production of chemicals derived from mitochondrial pyruvate (isobutanol) or acetyl-CoA (geraniol) [17]. While smaller compartment sizes and fusion deficits may be beneficial to the production of isobutanol, the same morphological alterations brought no benefit to geraniol production. This work shows that not all compartmentalized pathways respond equally to mitochondrial morphological changes. It also suggests that fermentative pathways might benefit from reduced mitochondrial size more than those relying on respiratory intermediates. This is likely due, at least in part, to mtDNA leakage and decreased respiratory capacity in fusion mutants.

While yeasts have proven to be versatile and hardy microbial factories for chemical production, mitochondrial morphology engineering in these fungi is still in its infancy. Taking advantage of the mitochondrial compartment and concentrating molecular pathways by reducing that compartment size is promising but there are several limitations to this tactic. Every gene that regulates mitochondrial morphology has additional roles within the cell; therefore, it is difficult to change mitochondrial size without impacting other cellular functions. This makes it challenging to determine whether the impact on chemical production is the result of mitochondrial morphology changes alone or due to other gene functions such as, for example, mitochondrial genome maintenance. Another limitation of this strategy is that it does not work for every chemical. Research suggests that constricting mitochondrial compartment size generally improves the production of chemicals produced from a fermentative pathway (using pyruvate as an intermediate) but has no meaningful impact on the production of chemicals that require respiratory intermediates such as acetyl-CoA [17]. Once the mitochondrial compartment is optimized, researchers face further challenges such as strain stability and balancing growth with chemical production for industrial applications.

Despite these limitations, mitochondrial morphology engineering for chemical production is a new and promising area of study. Coupling the inherent benefits of mitochondrial environments with changes in their compartment morphology can not only improve chemical production but might also help tailor compartmentalization strategies to desired chemical outputs. Each of these studies demonstrates the continued relevance of the yeast mitochondrion to engineering efforts and basic biology research.

## 5. Tools to Quantify Yeast Mitochondrial Morphology

When Richard Altmann first proposed the idea of mitochondria, calling them “bioblasts”, he described them as fundamental living units with both genetic and metabolic autonomy [105]. One of the earliest depictions of mitochondrial networks is a drawing published by Altmann in 1890 of two frog liver cells nestled next to each other, teaming with red webs of these “bioblasts”. Altmann’s ideas were met with such strong criticisms that Altmann was informally exiled from the scientific community, and it was not until decades later that the concept of mitochondria was legitimized by the scientific community [106,107,108]. Twenty-five years after Altmann drew his frog liver cells, the scientists Margaret Lewis and Warren Lewis, a couple conducting research together at Johns Hopkins Medical School, published the first study on mitochondrial morphology, diagraming and measuring morphology shifts by hand [109]. Since then, various techniques have been employed to measure changes in mitochondrial morphology and mitochondrial interactions with other organelles. Recent advances in microscopy have allowed imaging of cells with impressive resolution [110,111] and machine learning has enabled the detection and rendering of increasingly smaller structures [112,113].

In 1952, just as the popularization of mitochondrial study began to spread, two scientists working independently, George Palade and part-time artist Fritiof S. Sjöstrand, consecutively published the first high resolution images of mitochondria using electron microscopy [114,115,116]. Electron microscopy (EM) has persisted as a gold-standard method for direct visualization of mitochondria and their interactions with other organelles with nanometer-scale resolution. Furthermore, electron tomography now allows for 3D reconstructions of mito-ER contacts [117], although the process can be long and laborious. Transmission EM has been used to measure parameters such as the gap, width, and length of the interface between the ER and mitochondria [117]. Despite the unquestionable utility of these EM-based approaches, this analysis is limited to frozen samples and cannot be used to investigate living cells or mitochondrial dynamics.

In contrast to EM, fluorescence microscopy is perfectly suited to image live cells. This technique has been employed to assess mitochondrial morphology, dynamics, and mito-ER contacts in live [9] and fixed [118] samples. Assays based on the colocalization of ER and mitochondrial markers have been used to assess mitochondrial-ER contacts, although the resolution of many fluorescence microscopy techniques is not sufficient to determine all physiologically relevant interfaces. Confocal microscopy has made several great improvements over the past decade, including the ability to obtain super-resolution images from live cells expressing classical fluorophores [119,120] and newly introduced ultra-low-noise detectors that allow for quality imaging using orders of magnitude less laser power than required by standard spinning disk confocal microscopes [121,122].

Once high-quality images are achieved, the next hurdle is meaningful data analysis. There are many tools available for the analysis of biological imaging data (Table 2). A popular open-source tool that can provide colocalization measurements and quantitative analysis of z-stacks is Fiji (ImageJ) [21]. Other open-source programs such as MitoGraph [22] can render mitochondrial networks and other organelles, which can then be easily analyzed in the open-source workflow MitER [6]. Proprietary commercial options for image analysis include software like Imaris (Oxford Instruments, Oxon, UK) and Arivis (Arivis AG, Rostock, Germany). Both Arivis and Imaris provide tools for surface extraction and rendering from multichannel confocal images, which allows users to compute object-based intersections of subcellular structures. This can be used to look at inter-organelle contacts and their dimensions.

Though there have been recent advances in both imaging and analysis techniques, much remains to be uncovered as mitochondrial morphology continues to be central in studies relevant to human pathology and bioengineering. While several approaches are available to investigate subcellular dynamics, continued improvements such as more readily accessible rendering software and faster microscopy image acquisition at even lower laser power are critical if we are to further unravel the mysteries of mitochondrial morphology and dynamics.

## 6. Conclusions

The study of mitochondrial morphology in yeast has shed light on the complex processes that govern subcellular dynamics. Abnormal mitochondrial morphologies are associated with a wide variety of human diseases, and yeast models have been instrumental in understanding their underlying mechanisms. Furthermore, these aberrant mitochondrial phenotypes are being explored as potential assets in the development of strains for chemical production in the field of metabolic engineering.

As interest in mitochondrial morphology and mitochondrial compartment engineering increases, our means of classifying the different states that these organelles can adopt must advance accordingly. Techniques that allow accurate quantitative measurements of mitochondrial morphology and dynamics will be essential for this task. Some fragmentation patterns indicate increased cellular resistance in the tumor microenvironment, while others suggest respiratory deficits and predict neuronal apoptosis. Developing and standardizing metrics to meaningfully separate these fragmentation patterns would aid efforts to use screening of mitochondrial phenotypes as a method of early disease detection.

A standardized vocabulary for aberrant mitochondrial phenotypes would also be useful beyond healthcare. Precise classification of mitochondrial features has the potential to aid yeast strain engineering efforts to produce renewable energy and increase sustainable manufacturing. From the various metrics that could be analyzed, such as mitochondrial surface area, volume, and distribution, it could be possible to determine which compartment characteristics improve chemical production. Once identified, these features could be introduced in highly productive strains to further boost their productivity.

Since its inception, the study of yeast mitochondria has remained relevant across many fields of the life sciences and bioengineering. From identifying potential pathological processes to exploiting them for metabolic engineering, our understanding of yeast mitochondria is rapidly expanding. Precise and interdisciplinary metrics must be standardized to describe this organelle that sits firmly at the intersection between basic biological study, pathology, and bioengineering. Armed with this language, we can unify the mitochondrial dialog across fields.

## Figures and Tables

**Table 1:** Acronyms present in the paper along with their definitions.

Acronym	Full For
Aβ	Amyloid-β
AIM25	Activator of Mitochondrial Biogenesis 25
ATP	Adenosine Triphosphate
Caf4	Cdc28p-Associated Factor 4
Dnm1p	Dynamin-Related Protein 1 (Drp1p in humans)
Dnm2	Dynamin 2
DRP1	Dynamin-Related Protein 1
ER	Endoplasmic Reticulum
ERMES	ER-Mitochondria Encounter Structure
Fis1p	Mitochondrial Fission 1 Protein
Fzo1p	Fuzzy Onion 1 Protein
FUNDC1	FUN14 Domain Containing 1
GTP	Guanosine Triphosphate
LDs	Lipid Droplets
MAMs	Mitochondria-Associated ER Membranes
Mdm10p	Mitochondrial Distribution and Morphology 10 Protein
Mdm12p	Mitochondrial Distribution and Morphology 12 Protein
Mdm34p	Mitochondrial Distribution and Morphology 34 Protein
Mdv1p	Mitochondrial Division Protein 1
MGM1	Mitochondrial Genome Maintenance 1 Protein
Mgm1p	Mitochondrial Genome Maintenance 1 Protein
MFN1	Mitofusin 1
MFN2	Mitofusin 2
OMM	Outer Mitochondrial Membrane
OPA1	Optic Atrophy 1 Protein
PARL	Presenilin-Associated Rhomboid-Like Protein
PINK1	PTEN-Induced Putative Kinase 1
RAS	Rat Sarcoma Virus
ROS	Reactive Oxygen Species
SLC25A46	Solute Carrier Family 25 Member 46
TOM	Translocase of the Outer Membrane
Vps1p	Vacuolar Protein Sorting-Associated Protein 1
Ugo1p	Unknown Growth Protein 1

**Table 2:** Available bioanalysis tools. Table modified from A Hitchhiker’s guide through the bio-image analysis software universe [113].

Tool	Description	Citation
3D Slicer	An image processing software tailored for medical imaging, featuring 3D surface extraction, rendering, and analysis capabilities.	[123]
ANTs	Advanced Normalization Tools utilized for image registration, segmentation, and analysis, with a primary focus on neuroimaging.	[124]
Arivis Vision 4D	Image analysis software specialized in processing multi-channel 2D, 3D, and 4D microscopy data, with a particular emphasis on microscopy data.	(Arivis AG, Rostock, Germany)
Amira-Avizo	A versatile 2D–5D image processing, visualization, and analysis software, with customization options using Python and MATLAB.	(Thermo Fisher Scientific Inc., Waltham, MA, USA)
Bioformats	An image file format interoperability library that facilitates loading image data from various formats and vendors, supporting multiple image analysis software applications and programming environments.	[125]
Blender	A software with 3D surface rendering, modeling, and visualization capabilities. Additional features allow for Python scripting and video editing. Widely used in the design and arts community and increasingly employed for microscopy image data visualization.	[126]
BoneJ	A collection of image processing operations and ImageJ plugins tailored for skeletal/bone image analysis, with extensive usage in the soil, food, and materials science fields. Some tools are updated to work with ImageJ2.	[127]
CellPose	A deep-learning based segmentation algorithm specially designed for identifying biological structures such as cells and cell nuclei in microscopy images. Accessible as both a Python library and a standalone application, with CellPose plugins available for cellprofiler, qupath, and Fiji.	[128]
CellProfiler	An image analysis software application featuring a graphical user interface (GUI) for user-friendly configuration of standardized image analysis workflows, primarily targeting high-throughput microscopy imaging data of cells. Offers capabilities for extracting tabular image feature data, particularly in high-performance-computing environments.	[129]
DeconvolutionLab2	A collection of image deconvolution algorithms accessible as both a standalone command-line interface and a user-friendly ImageJ plugin.	[130]
Dragonfly	A software with an extensive set of tools for image processing, segmentation, and 3D visualization.	
Drishti	A visualization tool specialized for 3D pixel data, with additional functionality provided through segmentation and measurement tools.	[131]
Elastix	A standalone command-line tool primarily used for registration of 2D and 3D image data, based on the itk library. A python compatible interface, SimpleElastix, is also available.	[132]
Eman2	A software application focusing on cryoEM techniques, covering methods such as single particle analysis, cryo-electron tomography, and sub-tomogram averaging.	[133]
Fiji/ImageJ	An image analysis software based on ImageJ, offering a diverse collection of compatible plugins aimed at general-purpose image analysis in the life sciences. Scriptable using multiple programming languages compatible with the Java ecosystem and capable of handling big image data through integration of components like Imglib2 and BigDataViewer.	[21]
Huygens	An image processing software dedicated to deconvolution of 3D fluorescence microscopy images.	(Scientific Volume Imaging B.V., Hilversum, the Netherlands)
ilastik	An image analysis software offering machine learning for image segmentation, object classification, object tracking, and analysis of microscopy image data. ilastik classifiers can be used from Fiji and CellProfiler, and it supports execution on high-performance-computing clusters.	[134]
Imaris	An image processing and visualization software supporting 3D volume rendering and quantitative analysis. Through extra modules, it is interoperable with Fiji, Python, and MATLAB.	(Oxford Instruments, Oxon, UK)
IMOD	A collection of software for electron microscopy, offering image processing, modeling, and visualization capabilities.	[135]
ITK-SNAP	Software application designed for 3D medical imaging datasets, enabling segmentation and surface rendering.	[136]
Leica Application Suite X	Software for microscope control, image acquisition, visualization, and analysis with various modules for computations.	(Leica Microsystems GmbH, Wetzlar, Germany)
Matlab	A software environment for numeric computing, providing a multi-paradigm programming language and dedicated toolboxes.	(Mathworks, Natick, MA, USA)
Matplotlib	A scientific plotting and image visualization collection commonly used in the Python community.	[137]
Microscopy Image Browser	MATLAB-based software for advanced image processing, segmentation, quantification, and visualization of microscopy datasets.	[138]
MitER	A pipeline for Blender and R Studio for analysis of microscopy renderings with a focus on inter-organelle contacts in addition to organelle symmetry and distribution.	[6]
MitoGraph	A software for automated image processing method that offers full automation, specifically developed for calculating the volume of three-dimensional organelles and intracellular structures in live cells.	[22]
MorphoGraphX	Software for visualization and analysis of 4D datasets, particularly focused on organ growth from 4D live-imaging data.	[139]
NIS-Elements	Software for microscope control, image acquisition, visualization, and analysis with AI solutions for image restoration.	(Nikon, Tokyo, Japan)
ParaView	Software for surface data analysis and visualization that is based on the itk library.	[140]
OMERO	A research data management solution for microscopy image data, facilitating analysis of large amounts of imaging data.	[141]
opencv	A collection of image analysis components, focusing on computer vision algorithms and applications in microscopy.	(open computer vision)
R/RStudio	A programming language for statistical computing and plotting, commonly used for downstream analysis of image data.	[142]
RELION	Software package for cryo-EM structure determination, processing data from single particle or tomography experiments.	[143]
serialem	An acquisition software for various transmission electron microscopes, used for tomography and single-particle cryoEM.	[144]
smap	A MATLAB-based framework for single-molecule localization microscopy analysis, including molecule localization.	[145]
sr-tessler	Standalone software for quantitative analysis of localization-based super-resolution microscopy data.	[146]
tomviz	A software package for processing, visualization, and analysis of 3D tomographic data acquired with transmission electron microscopy.	[147]
zen	Software and components for microscope control, image acquisition, visualization, and analysis, integrating image analysis.	(Zeiss AG, Oberkochen, Germany)

Alternating blue and white color is used to facilitate line viewing.

## Data Availability

No new data were created or analyzed in this study. Data sharing is not applicable to this article.
